# Within trophic level shifts in collagen–carbonate stable carbon isotope spacing are propagated by diet and digestive physiology in large mammal herbivores

**DOI:** 10.1002/ece3.3786

**Published:** 2018-03-25

**Authors:** Daryl Codron, Marcus Clauss, Jacqueline Codron, Thomas Tütken

**Affiliations:** ^1^ Institut für Geowissenschaften AG für Angewandte und Analytische Paläontologie Johannes Gutenberg‐Universität Mainz Mainz Germany; ^2^ Florisbad Quaternary Research Department National Museum Bloemfontein South Africa; ^3^ Centre for Environmental Management University of the Free State Bloemfontein South Africa; ^4^ Clinic for Zoo Animals, Exotic Pets and Wildlife Vetsuisse Faculty University of Zürich Zürich Switzerland; ^5^ Mammalogy Department National Museum Bloemfontein South Africa

**Keywords:** browse, C_3_, C_4_, grass, methane, protein

## Abstract

Stable carbon isotope analyses of vertebrate hard tissues such as bones, teeth, and tusks provide information about animal diets in ecological, archeological, and paleontological contexts. There is debate about how carbon isotope compositions of collagen and apatite carbonate differ in terms of their relationship to diet, and to each other. We evaluated relationships between δ^13^C_collagen_ and δ^13^C_carbonate_ among free‐ranging southern African mammals to test predictions about the influences of dietary and physiological differences between species. Whereas the slopes of δ^13^C_collagen_–δ^13^C_carbonate_ relationships among carnivores are ≤1, herbivore δ^13^C_collagen_ increases with increasing dietary δ^13^C at a slower rate than does δ^13^C_carbonate_, resulting in regression slopes >1. This outcome is consistent with predictions that herbivore δ^13^C_collagen_ is biased against low protein diet components (^13^C‐enriched C_4_ grasses in these environments), and δ^13^C_carbonate_ is ^13^C‐enriched due to release of ^13^C‐depleted methane as a by‐product of microbial fermentation in the digestive tract. As methane emission is constrained by plant secondary metabolites in browse, the latter effect becomes more pronounced with higher levels of C_4_ grass in the diet. Increases in δ^13^C_carbonate_ are also larger in ruminants than nonruminants. Accordingly, we show that Δ^13^C_collagen_‐_carbonate_ spacing is not constant within herbivores, but increases by up to 5 ‰ across species with different diets and physiologies. Such large variation, often assumed to be negligible within trophic levels, clearly cannot be ignored in carbon isotope‐based diet reconstructions.

## 1 INTRODUCTION

Stable carbon isotope analysis is routinely used to reconstruct consumer resource use patterns in modern, archeological, and paleontological contexts (Ben‐David & Flaherty, 2012; Cerling & Harris, 1999; Cerling et al., 2015; Crawford, McDonald, & Bearhop, 2008; Hare & Sealy, 2013). Indeed, the approach provides arguably the most direct evidence for diet and habitat use among fossil animals. For this purpose, two phases of skeletal material may be analyzed: the collagen (protein) or carbonate (mineral apatite) phase, either from bone or from teeth (typically using dentine for the former, and tooth enamel for the latter). Whereas collagen typically degrades within a few thousand years after death, depending on climatic and environmental conditions, structurally bound carbonates in enamel undergo minimal postmortem diagenetic alteration and thus reflect lifetime carbon isotope compositions of animals from, in some cases, hundreds of millions of years ago (Bocherens, Drucker, Billiou, Patou‐Mathis, & Vandermeersch, 2005; Canoville, Thomas, & Chinsamy, 2014; Cerling, Harris, & Leakey, 2005; Fricke, Rogers, Backlund, Dwyer, & Echt, 2008; Lee‐Thorp, 2000; Sealy & van der Merwe, 1985; Tütken, 2011). The carbon in collagen, however, represents a different component of the diet than that of carbonates, and the two phases are thus not directly interchangeable sources of information (Ambrose & Norr, 1993; Krueger & Sullivan, 1984; Lee‐Thorp, Sealy, & van der Merwe, 1989). Concepts explaining these differences have been debated for some time, and resolution is needed to maximize the knowledge that can be gained from analysis of either material (Clementz, Fox‐Dobbs, Wheatley, Koch, & Doak, 2009; Howland et al., 2003).

The stable carbon isotope compositions of consumer collagen and carbonates (δ^13^C_collagen_ and δ^13^C_carbonate_) are usually more or less linearly related, although offsets from diet (diet‐tissue fractionation) differ (Ambrose & Norr, 1993; Howland et al., 2003; Lee‐Thorp et al., 1989; Passey et al., 2005; Tieszen & Fagre, 1993). In mammal herbivores, for example, δ^13^C_collagen_ is roughly 5 ‰ enriched in ^13^C relative to the diet, but this figure is much higher (~13 to 14 ‰) for δ^13^C_carbonate_, resulting in a difference (often referred to as Δ^13^C_collagen‐carbonate_ spacing) of ~9 ‰ in this animal group. Whereas body proteins, such as collagen, are synthesized mainly from dietary protein sources, structurally bound carbonate in the bioapatite derives from the bicarbonate pool of the body fluid and thus comprises a mixture of all dietary components, that is, proteins, carbohydrates, and lipids (Ambrose & Norr, 1993; Howland et al., 2003; McMahon, Fogel, Elsdon, & Thorrold, 2010; Tieszen & Fagre, 1993; Voigt, Rex, Michener, & Speakman, 2008). Hence, δ^13^C_carbonate_ values must reflect a weighted average of the whole diet. In cases where dietary components differ isotopically, consumer δ^13^C_collagen_ and δ^13^C_carbonate_ values may be less strongly related, or the relationship may deviate from linearity. Lipids in particular are ^13^C‐depleted relative to other biochemical carbon sources (Tieszen, Boutton, Tesdahl, & Slade, 1983), and therefore, a lipid‐rich diet is expected to lead to low δ^13^C_carbonate_ values. This observation has often been invoked to explain why Δ^13^C_collagen‐carbonate_ spacing in carnivores is only ~4 ‰, much lower than in herbivores, as carnivores consume a greater proportion of dietary lipids (Krueger & Sullivan, 1984; Lee‐Thorp et al., 1989; O'Connell & Hedges, 2017). Accordingly, Δ^13^C_collagen‐carbonate_ spacing has been used to infer trophic levels of fossil animals, including humans, recovered from sites where both materials retain lifetime isotopic compositions (e.g., Bocherens et al., 2017; Clementz et al., 2009).

An alternative explanation for the different δ^13^C_collagen_–δ^13^C_carbonate_ relationships across mammal trophic levels invokes physiological effects rather than effects of individual diet components (Hedges, 2003). One constraint to the diet‐based hypothesis is that lipids differ in δ^13^C from other biochemicals by too small an amount (~2 to 3‰) to account for the observed differences in Δ^13^C_collagen‐carbonate_ spacing between carnivores and herbivores (averaging ~5‰); Hedges (2003) published a series of mass balance simulations that supported this conclusion. The physiological hypothesis predicts relatively higher δ^13^C_carbonate_ values for herbivores than carnivores due to the large amounts of methane (CH_4_) produced as a by‐product of microbial fermentation in the gastrointestinal tract of herbivores, especially ruminants (Franz et al., 2010). Methane is substantially isotopically lighter (by ~40 to 50‰ in ^13^C) than other materials of biogenic origin (Klevenhusen et al., 2009; Metges, Kempe, & Schmidt, 1990; Schulze, Lohmeyer, & Giese, 1998); hence, carbon loss via CH_4_ is predicted to result in higher δ^13^C values remaining in the body nutrient pool, and thus in synthesized tissues. Further, some gaseous carbon released during fermentative digestion is in the form of ^13^C‐enriched CO_2_, some of which is resorbed into body fluids which may further enrich body fluid bicarbonate and, ultimately, δ^13^C_carbonate_ values (Hedges, 2003; Passey et al., 2005).

Early experimental tests with laboratory rodents showed that, whereas animal δ^13^C_carbonate_ is strongly related to δ^13^C_diet_, relationships between δ^13^C_collagen_ and δ^13^C_diet_ are weaker (Ambrose & Norr, 1993; Tieszen & Fagre, 1993). In those experiments, animal δ^13^C_collagen_ was more strongly related to δ^13^C values of dietary proteins, supporting the diet‐based model for observed spacing patterns. However, other controlled feeding experiments found differences in δ^13^C_diet‐carbonate_ spacing (fractionation) across species, suggesting a physiological effect (Passey et al., 2005; Warinner & Tuross, 2009). Still, it was argued that the comparisons between species from different studies reported by Warinner and Tuross (2009) were impaired because of differences in the complexity of species’ diets in the respective original studies (Froehle, Kellner, & Schoeninger, 2010). In particular, data for rodents were derived from animals fed complex pelleted diets, which are expected to be isotopically heterogeneous and could easily account for the different diet‐δ^13^C_carbonate_ and δ^13^C_collagen_–δ^13^C_carbonate_ relationships observed in rodents compared to experiments where animals had access to more natural diets (see also Codron, Sponheimer, et al., 2012). A recent experimental study found that Δ^13^C_protein‐carbonate_ spacing differed within a single *bird* species (hens) fed plant‐based (large spacing), meat‐based (smaller spacing), or omnivorous (intermediate spacing) diets (O'Connell & Hedges, 2017). Although these most recent experiments are consistent with a diet‐based explanation for patterns in δ^13^C_collagen_–δ^13^C_carbonate_ relationships, the authors noted that only hens on herbivorous diets reached isotopic equilibrium with their foods and so conclusions based on their estimates of spacing for meat based and omnivorous diets were treated with caution. Additionally, because hens also produce CH_4_ (Tsukahara & Ushida, 2000), physiologic effects of the different diets cannot be ruled out. The complicating factor in avian cecal fermentation is that in some species dietary fiber may not be the major substrate of fermentation and hence CH_4_ production, but uric acid transported retrogradely from the cloaca into the caeca (Frei, Ortmann, Kreuzer, Hatt, & Clauss, 2017).

Mammalian herbivores are a diverse group of animals with an array of diets and digestive physiologies that makes them suitable for testing diet‐ and physiology‐based predictions about δ^13^C_collagen_–δ^13^C_carbonate_ relationships in free‐ranging situations. Across species, diets are distributed along a browser‐grazer continuum, depending on whether diets are predominantly dicot or grass based (Clauss, Kaiser, & Hummel, 2008; Codron et al., 2007; Hofmann, 1989). Browse typically has a higher protein content than grass (Codron, Lee‐Thorp, Sponheimer & Codron, 2007a; Meissner, Zacharias, & O'Reagain, 1999; Van Soest, 1994); thus, we expect that δ^13^C_collagen_ should be biased toward browse‐based diets. In African Savannah environments, grasses are predominantly C_4_, with higher δ^13^C values than C_3_ browse. Hence, we expect differences between δ^13^C_collagen_ and δ^13^C_carbonate_ to emerge at the C_4_ (lower dietary protein) end of the scale, resulting in a higher rate of increase in δ^13^C_carbonate_ relative to δ^13^C_collagen_, that is, slopes >1 for this relationship, and shallower slopes for δ^13^C_collagen_ than for δ^13^C_carbonate_ when plotted against δ^13^C_diet_ (see Codron, Sponheimer, et al., 2012; Codron, Codron, et al., 2012). Similar patterns are also expected based on physiology, as grass‐based diets are associated with greater levels of methanogenesis because methane production is reduced by secondary plant compounds in browse such as tannins (Archimède et al., 2011; Jayanegara, Leiber, & Kreuzer, 2012; Staerfl, Zeitz, Kreuzer, & Soliva, 2012; White & Lawler, 2002). If CH_4_ production does play a role, however, we expect higher δ^13^C_carbonate_ values for ruminants compared with nonruminants such as hindgut fermenters (Cerling & Harris, 1999; Franz, Soliva, Kreuzer, Hummel, & Clauss, 2011; Franz et al., 2010). Carnivores, by contrast, consuming diets of less labile protein levels, and with less complex digestive systems, should have δ^13^C_collagen_–δ^13^C_carbonate_ relationships with slopes approaching 1. We tested these predictions using data from teeth and bones of 31 southern African Savannah herbivore species, all obtained from free‐ranging animals, and in comparison with 13 sympatric carnivore, three omnivore, and one primate species from the same environment, to ensure that predicted patterns are limited to herbivores only. We treat primates as a separate set of taxa because trophic level assignations of these taxa, especially baboons (*Papio ursinus*), are debatable, and because they likely have different physiological, anatomical, and behavioural traits distinguishing them from the “true” herbivores (mostly ungulates) included in the dataset.

## 2 METHODS

### 2.1 Data collection

The dataset used for this study was compiled from literature data on δ^13^C_collagen_ and/or δ^13^C_carbonate_ values in bones and/or teeth (dentine or enamel, respectively) of southern African mammals living in various biomes throughout southern Africa (Codron, Avenant, Wigley‐Coetsee, & Codron, 2017; Codron, Brink, Rossouw, & Clauss, 2008; Codron, Lee‐Thorp, Sponheimer, de Ruiter, & Codron, 2008; Codron, Brink, Rossouw, & Clauss, Codron, et al., 2008; Lee‐Thorp et al., 1989). The sample comprises individuals from the “lowveld” Savannah (*n *=* *142), “highveld Savannah” (*n *=* *160), “woodland” Savannah (*n *=* *58), grassland (*n *=* *168), and Kalahari (*n *=* *131) regions in the C_4_‐dominated interior of southern Africa including Malawi, as well as individuals from the Cape coastal (*n *=* *35) and succulent (*n *=* *19) regions, and the Namib Desert, Namibia (*n *=* *8) (see original source literature and online supplementary material Table S1 for more detail on the type of sample and their sample proveniences). Much of the Cape regions do not have a dominant C_4_ vegetation; hence, we only included specimens from localities where C_4_ grasslands do occur (see, e.g., Radloff, 2008). Of these specimens, δ^13^C_collagen_ data are available for 683, and 331 of those also have associated δ^13^C_carbonate_ data. A further 38 individuals have δ^13^C_carbonate_ but not δ^13^C_collagen_ data. The dataset comprises 58 species in total, categorized across four trophic groups: herbivores (*n *=* *40), carnivores (*n *=* *13), omnivores (*n *=* *3), and primates (*n *=* *1) (Table 1).

**Table 1:** Species included in this study, including average (median) δ^13^C_collagen_ and δ^13^C_carbonate_ values, Δ^13^C_collagen‐carbonate_ spacing, number of specimens (*n*), and interquartile ranges (IQR)

Species	%grass in diet	δ^13^C_collagen_		δ^13^C_carbonate_		Δ^13^C_collagen‐carbonate_	
		*n*	Median (IQR)	*n*	Median (IQR)	*n*	Median (IQR)
Herbivores: ruminants							
*Aepyceros melampus*	45	25	−15.6 (−16.8 to −14.8)	3	−8.2 (−8.3 to −6.8)	3	9.9 (8.5 to 10.2)
*Alcelaphus buselaphus*	75	23	−8.8 (−9.7 to −8.4)	11	1.0 (0.4 to 1.4)	11	10.2 (10.0 to 10.6)
*Alcelaphus lichtensteinii*	95	3	−6.2 (−6.5 to −6.2)	3	1.1 (0.1 to 1.4)	3	7.3 (6.3 to 7.8)
*Antidorcas marsupialis*	33	60	−18.3 (−20.3 to −16.7)	33	−9.6 (−11.1 to −7.9)	28	8.1 (6.1 to 10.1)
*Cephalophus natalensis*	1	1	−21.5 (−21.5 to −21.5)	1	−13.6 (−13.6 to −13.6)	1	7.9 (7.9 to 7.9)
*Connochaetes gnou*	81	55	−9.3 (−10.1 to −8.2)	12	0.6 (−0.1 to 1.9)	12	10.1 (9.3 to 10.7)
*Connochaetes taurinus*	88	59	−8.9 (−10.3 to −7.7)	15	1.3 (0.2 to 1.8)	15	10.0 (9.3 to 10.6)
*Damaliscus pygargus phillipsi*	90	17	−9.1 (−10.1 to −9.0)	11	1.9 (0.5 to 2.4)	11	11.3 (10.6 to 11.9)
*Giraffa camelopardalis*	0	7	−21.4 (−21.8 to −20.5)	5	−13.3 (−16.0 to −13.0)	5	8.4 (5.8 to 8.4)
*Hippotragus equinus*	85	4	−8.6 (−9.5 to −8.4)	3	1.6 (0.2 to 1.9)	3	10.8 (8.7 to 12.2)
*Hippotragus niger*	85	3	−7.3 (−8.5 to −7.2)	1	2.1 (2.1 to 2.1)	1	9.4 (9.4 to 9.4)
*Kobus ellipsiprymnus*	84	6	−8.2 (−8.7 to −7.6)	6	1.9 (1.2 to 2.5)	5	10.1 (8.6 to 11.1)
*Oryx gazella*	75	21	−10.9 (−12.2 to −9.0)	14	−1.7 (−2.6 to −1.1)	12	9.9 (8.4 to 10.6)
*Ourebia ourebi*	90	1	−13.7 (−13.7 to −13.7)	1	0.7 (0.7 to 0.7)	1	14.4 (14.4 to 14.4)
*Raphicerus campestris*	34	10	−20.3 (−21.4 to −19.2)	9	−11.9 (−12.8 to −10.8)	9	8.6 (8.1 to 10.4)
*Raphicerus melanotis*	30	3	−20.3 (−20.8 to −20.3)	3	−14.7 (−14.9 to −14.3)	1	5.6 (5.6 to 5.6)
*Redunca arundinum*	94	13	−8.1 (−9.9 to −7.4)	10	1.4 (−0.1 to 2.6)	10	10.3 (8.3 to 10.9)
*Sylvicapra grimmia*	12	17	−20.9 (−21.7 to −20.1)	15	−13.8 (−14.2 to −12.3)	12	7.2 (5.7 to 7.7)
*Syncerus caffer*	78	15	−9.3 (−13.2 to −9.0)	9	1.0 (−0.3 to 1.5)	9	10.2 (8.7 to 11.4)
*Tragelaphus angasii*	20	10	−16.7 (−17.6 to −16.4)	9	−10.1 (−11.3 to −9.5)	9	6.6 (6.1 to 7.2)
*Tragelaphus oryx*	50	21	−19.4 (−20.8 to −18.4)	11	−10.6 (−11.8 to −10.4)	11	8.4 (7.9 to 9.1)
*Tragelaphus scriptus*	10	17	−22.3 (−23.1 to −21.1)	20	−14.4 (−15.6 to −13.9)	13	6.9 (6.0 to 8.2)
*Tragelaphus strepsiceros*	15	38	−20.6 (−21.9 to −19.7)	14	−11.7 (−12.8 to −10.0)	10	7.4 (6.1 to 8.3)
Herbivores: non‐ruminants							
*Ceratotherium simum*	98	9	−8.5 (−9.0 to −6.1)	3	0.5 (−5.4 to 0.8)	3	9.4 (8.6 to 9.5)
*Diceros bicornis*	5	6	−21.5 (−21.7 to −21.4)	3	−14.7 (−15.2 to −13.7)	3	7.5 (6.6 to 7.9)
*Equus quagga*	90	51	−10.4 (−11.6 to −9.6)	14	0.6 (0.0 to 1.2)	14	10.0 (7.8 to 11.3)
*Equus zebra zebra*	95	8	−10.7 (−11.7 to −9.5)	1	−2.0 (−2.0 to −2.0)	1	7.7 (7.7 to 7.7)
*Hippopotamus amphibius*	95	8	−11.2 (−13.3 to −10.3)	7	−4.3 (−5.2 to −2.8)	7	8.3 (7.7 to 9.2)
*Phacochoerus aethiopicus*	95	2	−12.5 (−12.8 to −12.1)	2	−6.2 (−6.7 to −5.6)	2	6.3 (6.2 to 6.5)
*Phacochoerus africanus*	95	43	−11.0 (−11.6 to −10.3)	12	−0.4 (−2.0 to −0.2)	11	8.8 (8.1 to 9.3)
*Procavia capensis*	5	10	−20.2 (−21.2 to −19.1)	10	−9.9 (−12.0 to −8.9)	10	9.5 (9.2 to 10.1)
Carnivores							
*Canis mesomelas*		4	−16.0 (−18.2 to −13.3)	4	−9.4 (−12.6 to −6.5)	4	5.4 (4.9 to 6.3)
*Caracal caracal*		8	−15.7 (−18.7 to −13.8)	6	−11.2 (−14.3 to −8.3)	6	4.8 (4.6 to 5.3)
*Crocuta crocuta*		1	−10.9 (−10.9 to −10.9)	3	−4.3 (−5.8 to −4.3)	1	3.5 (3.5 to 3.5)
*Felis sylvestris*		3	−13.6 (−16.3 to −12.0)	3	−8.8 (−11.7 to −6.9)	3	4.8 (4.6 to 5.1)
*Genetta genetta*		1	−13.2 (−13.2 to −13.2)	1	−8.6 (−8.6 to −8.6)	1	4.6 (4.6 to 4.6)
*Leptailurus serval*		2	−13.9 (−14.1 to −13.7)	2	−11.1 (−11.1 to −11.0)	2	2.9 (2.6 to 3.1)
*Lycaon pictus*		3	−11.7 (−11.9 to −11.7)	3	−6.4 (−7.2 to −5.9)	3	5.3 (4.7 to 5.8)
*Otocyon megalotis*		6	−17.5 (−19.5 to −15.3)	6	−13.3 (−16.0 to −10.3)	6	4.8 (4.0 to 5.0)
*Panthera leo*		14	−10.7 (−11.7 to −9.5)	13	−5.9 (−7.7 to −2.8)	10	3.6 (3.3 to 5.0)
*Panthera pardus*		14	−17.7 (−18.9 to −12.3)	11	−12.8 (−14.1 to −8.3)	11	4.3 (3.7 to 5.0)
*Parahyaena brunnea*		8	−11.0 (−11.9 to −9.7)	8	−7.1 (−8.7 to −5.7)	7	3.6 (2.2 to 4.1)
*Proteles cristata*		1	−13.3 (−13.3 to −13.3)	1	−8.2 (−8.2 to −8.2)	1	5.1 (5.1 to 5.1)
*Vulpes chama*		4	−14.0 (−14.9 to −12.6)	4	−7.7 (−9.3 to −7.0)	4	5.2 (3.9 to 6.2)
Omnivores							
*Hystrix africaeaustralis*		3	−20.7 (−20.8 to −20.7)	3	−15.6 (−16.1 to −14.6)	3	5.0 (4.7 to 6.1)
*Potamochoerus porcus*		2	−19.1 (−19.7 to −18.5)	2	−12.7 (−14.6 to −10.8)	2	6.4 (5.1 to 7.7)
*Xerus inaurus*		1	−11.2 (−11.2 to −11.2)	1	−5.0 (−5.0 to −5.0)	1	6.3 (6.3 to 6.3)
Primates							
*Papio ursinus*		29	−18.5 (−19.7 to −17.2)	23	−12.3 (−12.8 to −11.7)	20	5.5 (4.8 to 6.1)

%grass in diet of herbivores are averages taken from the literature, estimated from field observations (Gagnon & Chew, 2000; Owen‐Smith, 2013; Skinner & Chimimba, 2005).

### 2.2 Data analysis

The data used includes carbon isotope compositions of both teeth (dentine collagen and enamel carbonate) and bone (collagen and apatite carbonate). These two tissues may differ in carbon isotope compositions (Melin et al., 2014; Warinner & Tuross, 2009). To ensure the data could be treated as a single sample, we compared δ^13^C_collagen_ and δ^13^C_carbonate_ values from teeth and bones of each species (where possible) using paired sample *t‐*tests. Also, because specimens in the dataset were collected at different times, we subtracted 2‰ from δ^13^C values of individuals that died before 1950 to account for atmospheric changes in δ^13^CO_2_ (Francey et al., 1999).

To compare relationships between δ^13^C_carbonate_ and δ^13^C_collagen_ across trophic groups (herbivores, carnivores, omnivores, and primates), we used reduced major axis (RMA) regression models. We treated δ^13^C_carbonate_ as the “response” (*y*‐axis) variable to be consistent with other approaches in the literature (e.g., Lee‐Thorp et al., 1989), and because previous studies have predicted that physiology mainly affects δ^13^C_carbonate_ rather than δ^13^C_collagen_ (Hedges, 2003; O'Connell & Hedges, 2017; Passey et al., 2005). These regression models were applied to each trophic group (herbivores, carnivores, omnivores, and primates) separately, using data for all individuals, regardless of taxon, for which both δ^13^C_carbonate_ and δ^13^C_collagen_ data are available. We then repeated the analyses using species averages (medians) to ensure the results were not bias by inclusion of multiple taxa, and different sample sizes for each taxon; in this instance, omnivores (*n *=* *3) and primates (*n *=* *1) were necessarily omitted. Further, because both physiology and diet, both of which are predicted to influence animal isotope compositions, are at least in part dependent on species’ phylogenetic affiliations, we applied a phylogenetically constrained RMA to these analyses. We used a pruned version of a mammalian supertree (Fritz, Bininda‐Emonds, & Purvis, 2009), which was then correlated to our data using the phyl.RMA function of the R package phytools (Revell, 2012).

The above analyses make the explicit assumption that animal δ^13^C_carbonate_ values are dependent on δ^13^C_collagen_ values. While this approach is common in the literature, and provides insights into patterns of Δ^13^C_carbonate‐collagen_ spacing, it does not explicitly test for different effects on either dataset. As our main hypothesis is that dietary differences among herbivores, that is, browsing vs grazing, affect δ^13^C_carbonate_ and δ^13^C_collagen_ differently, we tested models regressing either dataset on the percentage grass in the natural diet of each species (herbivores only). The latter were taken from the literature, using averages derived from field studies (Gagnon & Chew, 2000; Owen‐Smith, 2013; Skinner & Chimimba, 2005). Although carbon isotope studies have revealed differences between estimated %C_4_ grass in the diet when compared with data from field studies for several taxa (Cerling, Harris, & Passey, 2003; Codron et al., 2007; Sponheimer et al., 2003), due in part to differences in habitats and spatiotemporal scales investigated, the broad agreement between the two approaches means that published field data provides an independent dataset that is well‐suited to our purposes. We used GLMs to assess relationships between δ^13^C_carbonate_ and δ^13^C_collagen_ with %grass in each species’ natural diet separately. We also included a term describing digestion type to test for differences among ruminants (23 species) and nonruminants (8 species), and also the interaction of digestion type with %grass in the diet. We selected the best fit models from these various combinations based on the small sample corrected Akaike's Information Criterion, AIC_c_, assuming models with ΔAIC_c_ (AIC_c_ for each model, in turn, minus the lowest AIC_c_ among all candidate models) <2 are best supported by the data (Burnham & Anderson, 2001, 2002). Phylogenetically controlled analyses of these models were performed using phylogenetic least squares regression (PGLS), employing maximum‐likelihood estimation of phylogenetic signal (lambda), in the package “caper” (Orme et al., 2013). All analyses were performed in R 3.4.2 (R Core Team, 2015).

## 3 RESULTS

### 3.1 Comparison between teeth and bone

The δ^13^C_collagen_ of tooth dentine collagen was, on average, 1.3‰ ± 1.97 *SD* lower than that of bone collagen. Although the difference is statistically significant (*t*_29_ = −3.560, *p *<* *.01), it is of fairly small magnitude in terms of degree of error (~1 to 2‰) in diet reconstructions in southern African Savannah systems (Codron, Lee‐Thorp, Sponheimer, & Codron, 2007b). The δ^13^C_carbonate_ of tooth enamel and bone apatite carbonate differed by an even smaller magnitude (0.8‰ ± 2.15 *SD* higher in tooth enamel), and in this case, the difference was not significant (*t*_24_ = 1.783, *p *=* *.088). Moreover, there was no consistent trend in terms of the difference between teeth and bone for either δ^13^C dataset, in that the differences were not correlated to δ^13^C (Pearson's *r *=* *.095, *t*_28_ = 0.507, *p *=* *.616 for δ^13^C_collagen_; *r *=* *.279, *t*_22_ = 1.361, *p *=* *.187 for δ^13^C_carbonate_). Thus, combining data from teeth and bones does not increase overall variability in our dataset, nor does it contribute to the strength of any relationships described below, and it is therefore appropriate to combine these data into a single dataset of collagen and carbonate for this study.

### 3.2 Individuals with both collagen and carbonate data

**Figure 1:**
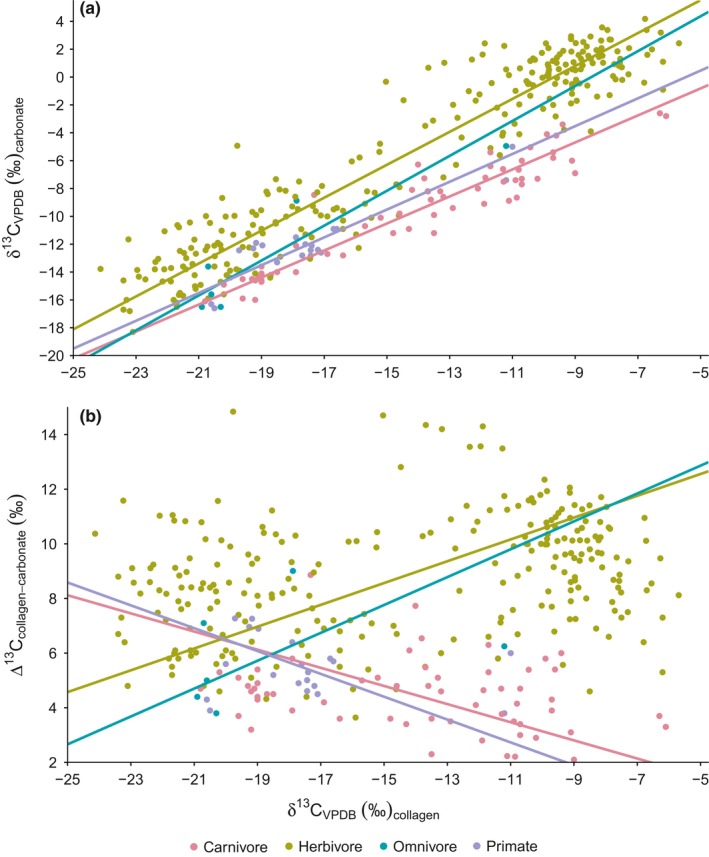
Relationships of δ^13^C_carbonate_ (a) and Δ^13^C_collagen‐carbonate_ spacing (b) with δ^13^C_collagen_ among southern African mammals. Each data point represents a single individual for which both δ^13^C_carbonate_ and δ^13^C_collagen_ data are available. Fit lines are reduced major axis regressions

There was a strong positive relationship between δ^13^C_carbonate_ and δ^13^C_collagen_ in all four trophic groups (Figure 1a). Herbivores showed a higher intercept (10.692) than carnivores (4.035), reflecting the higher Δ^13^C_carbonate‐collagen_ spacing expected for the former group (Table 2). The intercept for omnivores was similar to that of herbivores (10.631), but the sample is small (*n *=* *6 individuals) and so 95% confidence intervals around this estimate were broad and overlapped with both herbivores and carnivores. The intercept for primates was similar to that of carnivores (5.458).

**Table 2:** Parameter estimates (means with 95% CI in parentheses) from reduced major axis (RMA) regressions for relationships between δ^13^C_collagen_ and δ^13^C_carbonate_ (the latter treated as the “response” variable) among southern African mammals, by trophic group. Parameters are presented as means, with 95% confidence intervals in parentheses

Group	*n*	*r* ^2^	*p*	Intercept	Slope	Lambda
All individuals						
Herbivore	246	.8960	<.0001	11.4303 (10.6915 to 12.1692)	1.1819 (1.1348 to 1.2309)	
Carnivore	59	.8904	<.0001	4.0351 (2.8007 to 5.2696)	0.9700 (0.8886 to 1.0589)	
Omnivore	6	.8495	<.01	10.6308 (−2.1408 to 23.4024)	1.2530 (0.7484 to 2.0978)	
Primate	20	.8322	<.0001	5.4580 (1.8584 to 9.0576)	0.9982 (0.816 to 1.221)	
Species averages (medians)						
Herbivore	31	.9335	<.0001	11.0933 (9.3921 to 12.7945)	1.1670 (1.0582 to 1.2869)	
Carnivore	13	.7272	<.001	3.1508 (−1.1924 to 7.494)	0.8923 (0.6351 to 1.2537)	
Phylogenetically‐constrained RMA						
Herbivore	31	.9335	<.0001	11.0933	1.1670	0.0001
Carnivore	13	.7272	<.0001	3.1509	0.8923	0.0001

However, it was not only the intercepts of these regressions that differed across trophic groups, but the slopes as well. Whereas carnivores, omnivores, and primates all had slopes ≤1, the slope for herbivores was significantly >1 (95% CI 1.135 to 1.231; Table 2). Accordingly, whereas Δ^13^C_carbonate‐collagen_ spacing in carnivores (and primates) was negatively related to δ^13^C_collagen_, these relationships were positive for herbivores (and also for omnivores, although the small sample for this group makes it difficult to properly validate this finding; Figure 1b). Although regressions of spacing on δ^13^C values for either tissue are spurious (because the δ^13^C value also appears in the estimate of spacing), an RMA applied to these data indicates that the slope for herbivores is significantly >1 (95% CI 0.354 to 0.450) and that of carnivores is <1 (−0.428 to −0.258). In both cases (Figure 1a,b), the high slope for herbivores reflects our prediction of a more than proportional increase in herbivore δ^13^C_carbonate_ values in response to increasing dietary δ^13^C.

### 3.3 Species‐level analysis

**Figure 2:**
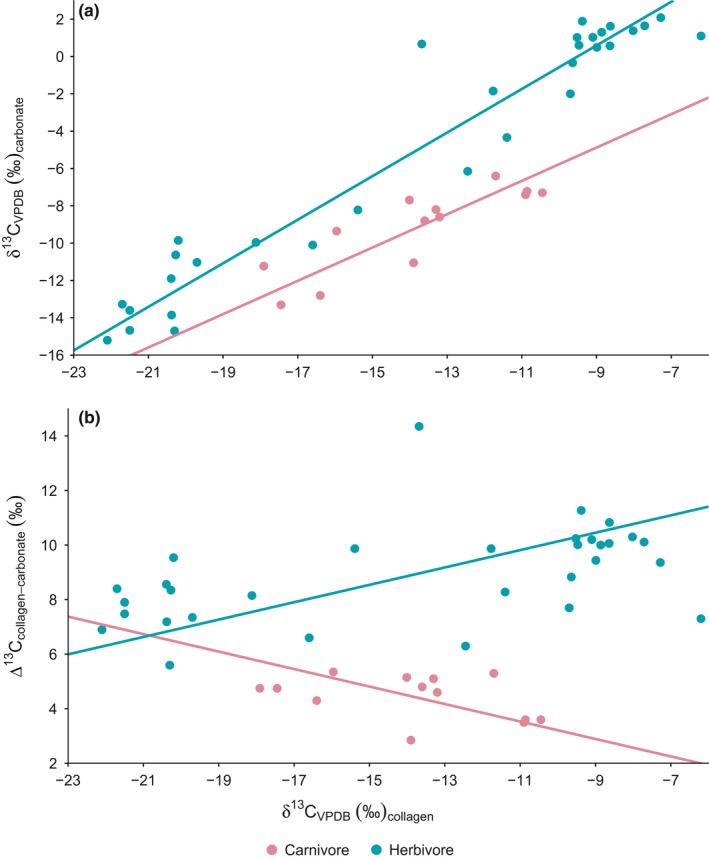
Relationships of δ^13^C_carbonate_ (a) and Δ^13^C_collagen‐carbonate_ spacing (b) with δ^13^C_collagen_ across southern African mammal species. Each data point represents the medians of a species. Fit lines are reduced major axis regressions

Repeating the above analysis at species level, that is, using species’ median δ^13^C_collagen_ and δ^13^C_carbonate_ values, yielded similar results (Figure 2; note that omnivores and primates are not included in these analyses). Herbivores had a higher intercept than carnivores, and whereas the slope for herbivores was significantly >1 (95% CI 1.058 to 1.287), that of carnivores was ≤1 (Table 2). Similarly, herbivores again showed a positive relationship between Δ^13^C_carbonate‐collagen_ spacing and δ^13^C_collagen_ (95% CI for slope 0.230 to 0.442), whereas in carnivores, the relationship was negative, albeit not significant (95% CI for slope −0.569 to −0.180, *p *=* *.171).

The phylogenetically constrained analysis of these regressions revealed a very weak phylogenetic signal in the data (lambda approaching 0; Table 2), and hence similar patterns, with a slope >1 for herbivores, and <1 for carnivores.

### 3.4 Relationships with field data

**Figure 3:**
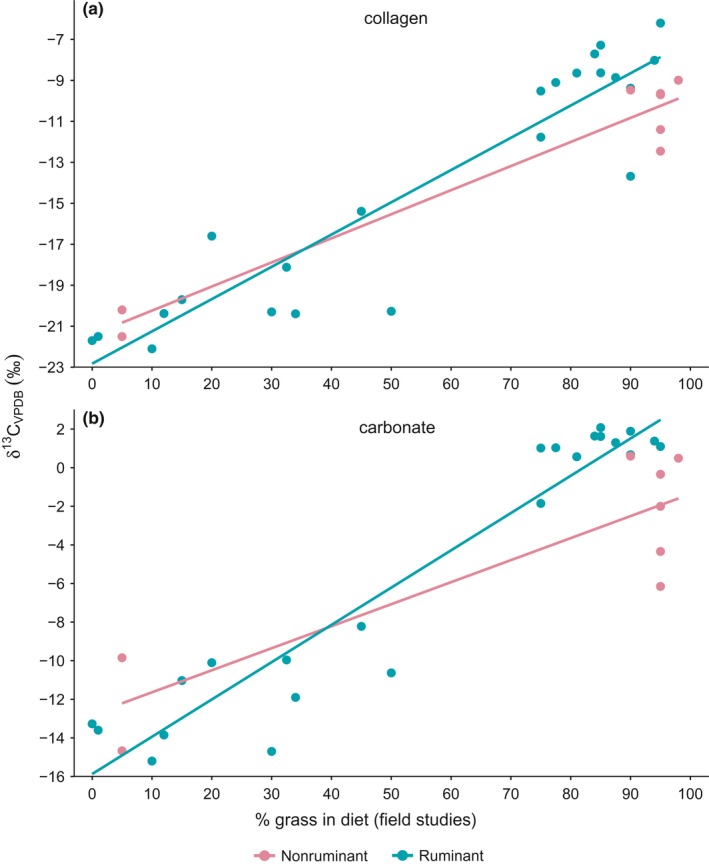
Relationships between δ^13^C_collagen_ (a) and δ^13^C_carbonate_ (b) among southern African herbivores with the predicted percentage of C_4_ grass in each species’ diet, showing differences between ruminant and nonruminant taxa

Relationships with (presumed) levels of C_4_ grass in species’ diets (using literature data derived from field observations) were investigated to explicitly test for different influences on δ^13^C_collagen_ and δ^13^C_carbonate_ values among herbivores. Both sets of data were significantly and strongly related to %grass in the diet, regardless of whether phylogenetic history was accounted for (Table 3). In both cases (i.e., GLM and PGLS), models that took into account differences in digestive strategy (ruminant versus nonruminant) had no influence on the response of δ^13^C_collagen_ to diet (ΔAIC_*c*_ for all models <2, and see Figure 3a). However, the relationship between %grass in the diet and δ^13^C_carbonate_ did differ across the two digestive groups (Figure 3b). Actually, in the latter instance, models including the interaction between diet and digestion type were clearly best supported (ΔAIC_c_ for other models >6), indicating that both the intercept and slope for ruminants was significantly higher than those for nonruminants. In other words, these results predict not only higher δ^13^C_carbonate_ values among ruminants than nonruminants, but also a faster rate of increase in response to increasing levels of grass in the diet.

**Table 3:** Effects of % C_4_ grass in the natural diet on δ^13^C_collagen_, δ^13^C_carbonate_, and δ^13^C_collagen‐carbonate_ spacing in 31 species of Savannah herbivores, based on general linear models (GLMs) and phylogenetic least squares regressions (PGLS). The models also test for an influence of digestion type (GIT, i.e., ruminant or nonruminant) on these relationships

Model	GLM				PGLS				
	*K*	AIC_*c*_	ΔAIC_c_	*r* ^2^(adj)	*K*	AIC_c_	ΔAIC_c_	*r* ^2^	Lambda
δ^13^C_collagen_									
%grass**** + GIT + interaction	5	139.82	0.00	.8666	4	136.96	0.00	.8799	0.0001
%grass****	3	139.93	0.11	.8561	2	137.47	0.51	.8561	0.0001
%grass**** + GIT	4	140.43	0.60	.8561	3	137.78	0.82	.8657	0.0001
δ^13^C_carbonate_									
%grass**** + GIT* + interaction**	5	147.09	0.00	.8762	4	144.23	0.00	.8886	0.0001
%grass**** + GIT	4	153.91	6.83	.8368	3	150.78	6.55	.8497	0.6419
%grass****	3	155.37	8.28	.8261	2	150.31	6.08	.8363	0.7316
Δ^13^C_collagen‐carbonate_ spacing									
%grass** + GIT + interaction*	5	116.45	0.00	.3808	4	113.59	0.00	.4427	0.0001
%grass** + GIT	4	120.68	4.23	.2495	3	118.03	4.44	.2995	0.0001
%grass**	3	120.99	4.54	.2293	2	118.53	4.94	.2293	0.0001

*r* ^2^(adj) = *r* ^2^ adjusted for additional parameters; ****p < .0001; ***p < .001; **p < .01; *p < .05 (model structure and significance of each effect only shown in one column because results for GLM and PGLS are virtually identical).

## 4 DISCUSSION

Relationships between mammal δ^13^C_carbonate_ and δ^13^C_collagen_ values differ across trophic levels, not only in terms of the well‐known differences in Δ^13^C_collagen‐carbonate_ spacing (i.e., lower intercepts for carnivores than herbivores) but also in their slopes. Whereas slopes for carnivores approximate or include 1, and Δ^13^C_collagen‐carbonate_ spacing remains more‐or‐less constant across the full range of diets, slopes >1 occur in herbivores and hence Δ^13^C_collagen‐carbonate_ spacing increases with increases levels of C_4_ grass intake. This result is consistent with the expectation of a bias toward ^13^C‐depleted C_3_ foods in δ^13^C_collagen_ due to the higher protein content (two‐ to threefold) of C_3_ browse than C_4_ grass (cf. Howland et al., 2003; Jim, Ambrose, & Evershed, 2004; Tieszen & Fagre, 1993), and with predicted increases in δ^13^C_carbonate_ due to higher levels of ^13^C‐depleted methane production associated with grass‐rich diets (Cerling & Harris, 1999).

We cannot separate diet from physiological effects based on these data and analyses alone, but it is likely that both play a role in determining isotope compositions of collagen and carbonate, and the spacing between them. The influence of physiology is, however, supported by the different responses observed between ruminants and nonruminants in terms of δ^13^C_carbonate_ in relation to %grass intake (Figure 3). The steeper slope for ruminants is not surprising as they produce more CH_4_ than large hindgut fermenters such as equids (Franz et al., 2010, 2011), and factors that result in higher rates of methane production would therefore affect ruminants to a greater extent. Nonruminant foregut fermenters (hippopotamus, *Hippopotamus amphibius*, in our dataset) may produce CH_4_ in amounts intermediate between ruminants and hindgut fermenters, as previously shown for the pygmy hippopotamus *Hexaprotodon liberiensis*, and collared peccary *Pecari tajacu* (Vendl et al., 2016). However, hippopotamus δ^13^C_carbonate_ values presented here are within range of other nonruminant grazers in our dataset.

Previous studies have found similar Δ^13^C_collagen‐carbonate_ spacings across trophic levels as observed here, but generally predicted this spacing to be constant within trophic levels (Clementz, 2012; Clementz et al., 2009). Our results suggest this is not the case, at least for mammal herbivores, in which variation in food quality and digestive physiology across species alter δ^13^C_collagen_–δ^13^C_carbonate_ relationships in such a way that spacing does vary, and this variation can be of considerable magnitude. Considering only taxa with *n *>* *5 individuals, the lowest average (median) Δ^13^C_collagen‐carbonate_ spacing in our data occurred in the nyala *Tragelaphus angasii* (*n *=* *9, median = 6.6‰, min–max range = 5.4 to 9.5‰), and the largest in blesbok *Damaliscus pygargus phillipsi* (*n *=* *11, median = 11.3 ‰, min–max range = 10.6 to 11.9‰). There has been some debate in the literature about whether analysis of collagen or carbonate provides a better representation of an animal's diet. The wide range of spacing across species observed in this study—about one‐third of the total range of δ^13^C values in the system within a single trophic level—coupled with the fact we cannot yet differentiate specific effects, implies that, wherever possible, both materials should be analyzed, in agreement with suggestions of Clementz et al. (2009) and Froehle et al. (2010). Alternatively, a correction factor/s based on regression models similar to those used in our study could be applied to either source of data, but the lack of independent data for diets in many cases would limit this possibility. For instance, while our use of literature data for %grass in species’ diets could be used to parameterize models for isotopic fractionation, this approach would assume that species’ diets are fixed and unvarying, which they are not (Owen‐Smith, 1997; du Toit, 2003).

Actually, within‐species variation in diet raises an exciting possibility for future exploration: both mechanisms proposed here, that is, lower protein intake and higher CH_4_ production on grass‐rich diets, operate within‐ as well as between‐species (Staerfl et al., 2012; Van Soest, 1994; White & Lawler, 2002). We do not have sufficient data for any one taxon across a range of diets, for example, sampled across several habitats and/or seasons, to test predictions at the intraspecific level. However, a previous study based on serial isotope analysis of ivory of African elephants, *Loxodonta africana*, found that δ^13^C_carbonate_ series in this species were less variable than δ^13^C_collagen_ series extracted over the same time frames and time scales, that is, seasonal over several decades within individuals (Codron, Codron, et al., 2012). Those results are consistent with dietary protein effects, as elephants are well‐known to switch from protein‐rich browse‐based diets during dry periods to grass‐rich diets during rainy seasons (Codron et al., 2011; Owen‐Smith, 2013; Pretorius et al., 2012). But those results do not unequivocally support a diet effect, as the difference may reflect that carbonate has a slower turnover rate than collagen and thus did not capture the same extent of seasonal variability in diet (see Codron, Codron, et al., 2012).

One factor not considered in our study is whether C_3_ browse and C_4_ grass differ with respect to inherent δ^13^C variability. There is some evidence that grass δ^13^C_lipid_ differs from δ^13^C_carbohydrate_ and δ^13^C_protein_ by a larger amount than in dicots (Dungait, Docherty, Straker, & Evershed, 2008). Such differences, if they are found to persist, could easily account for differences in Δ^13^C_collagen‐carbonate_ spacing between trophic levels, and even within herbivores. Empirical data for compound‐specific isotope compositions of Savannah plants are needed. More so, however, controlled experiments like those of O'Connell and Hedges (2017), which explicitly test for diet‐linked physiological effects on spacing, are needed. The drawback is that such experiments are necessarily long term, given the time needed for bone to be remodeled and become equilibrated with an experimental diet/s. Nonetheless, advances in this field, and improved accuracy in paleodiet reconstructions, require that researchers trade‐off short‐ for long‐term gains.

## CONFLICT OF INTEREST

None declared.

## AUTHORS’ CONTRIBUTIONS

DC conceived the approach and design of the study, DC and JC conducted fieldwork and laboratory work, MC developed ideas relating to differences in methanogenesis between species and diets, TT collaborated in geochemical principles, implications for archeology/paleoecology, and insights from recent literature, DC and JC wrote the manuscript. All authors contributed critically to the drafts and gave final approval for submission.
